# Analysis of the influencing factors on fine-needle aspiration biopsy results of the thyroid

**DOI:** 10.3389/fsurg.2022.907086

**Published:** 2022-09-05

**Authors:** Chun-Yan Wang, Yan Zhou, Yu-Yuan Ren, Yu-Shuang Luan, Zhong-Cai Jiang, Zhi-Xing Wang

**Affiliations:** ^1^ Department of Endocrinology, Aviation General Hospital, Beijing, China; ^2^ Department of Pathology, Aviation General Hospital, Beijing, China; ^3^ Department of Ultrasonography, Aviation General Hospital, Beijing, China

**Keywords:** thyroid nodule, biopsy, needle, capillary, ultrasonography, cytology

## Abstract

**Objective:**

The objective of the study is to analyze the influencing factors on the results of thyroid fine-needle aspiration biopsy (FNAB).

**Method:**

A total of 339 patients who underwent FNAB in our hospital from December 2018 to July 2021 were retrospectively selected. The patients were chosen according to the gender ratio, age, and thyroid ultrasound characteristics and were divided into three groups: (1) a 22G needle vacuum aspiration group (Group 1, *n* = 85), (2) a 22G biopsy needle non-vacuum aspiration group (Group 2, *n* = 50), and (3) a 25G biopsy needle non-vacuum aspiration group (Group 3, *n* = 204). Patients in these groups were evaluated for determining the FNAB dissatisfaction rate of pathological samples. A bivariate regression analysis of independent risk factors related to the unsatisfactory pathological diagnosis of samples was performed.

**Results:**

The specimen dissatisfaction rates of the three groups were 22/85 (25.9%), 15/50 (30%), and 18/186 (9.7%), respectively. The overall sample dissatisfaction rate was 55/339 (16.2%), and the sample satisfaction rate of Group 3 was higher than that of Groups 1 and 2 (*P *< 0.05). Logistic bivariate regression analysis showed that the age of the patients and the capillary sampling needles and aspiration methods were two independent risk factors for determining the dissatisfaction rate of FNAB pathological samples.

**Conclusion:**

A 25G capillary sampling aspiration biopsy needle was selected to perform FNAB in thyroid nodules without vacuum aspiration, which could effectively improve the accuracy of FNAB results with a high specimen satisfaction rate.

## Introduction

Thyroid nodules are one of the most common diseases encountered by the surgeons of the endocrinology and general surgery departments, of which malignant lesions account for a small proportion [1–3]. A large amount of data in evidence-based medicine have shown that fine-needle aspiration biopsy (FNAB) is a safe and minimally invasive diagnostic technique and is considered to be the current gold standard for distinguishing benign and malignant thyroid nodules [4–6]. FNAB can improve the accuracy of a preoperative diagnosis of thyroid nodules by up to 80%–90% [1], but it still cannot make a clear diagnosis in certain patients. Pathologists were unable to make a clinical diagnosis based on these samples due to an inadequate number of cells or difficulty in identifying cells in certain samples during the process of FNAB sampling. In this study, unsatisfactory FNAB samples by cytological diagnosis were selected to analyze the influencing factors on FNAB results of the thyroid and to improve the success rate of thyroid capillary sampling, thus providing favorable evidence for pathological diagnosis of thyroid FNAB.

## Materials and methods

### Subjects

This study was a retrospective analysis of 339 patients (79 male and 260 female) who underwent FNAB at the Aviation General Hospital in Beijing, China, from December 2018 to July 2021, with an average age of 47.38 ± 13.48 years. There were 55 cases of unsatisfactory pathologic results to analyze the influencing factors on FNAB results of the thyroid.

### Methods

After collecting the thyroid ultrasound characteristics of the patients, including nodule size, blood flow, type, and boundary, as well as needles and aspiration methods used for thyroid FNAB, all patients were divided into three groups according to the different types of needles and aspiration methods used in FNAB. Group 1: a 22G biopsy needle (outside diameter 0.7 mm) vacuum aspiration group (*n* = 85); Group 2: a 22G needle (outside diameter 0.7 mm) capillary sampling aspiration group (*n* = 50); Group 3: a 25G biopsy needle (outside diameter 0.5 mm) capillary sampling aspiration group (*n* = 204). The three groups were compared for determining the satisfaction rate of thyroid cell samples. All the enrolled patients were evaluated for thyroid nodule risk by thyroid specialists who would determine whether to perform FNAB according to the inclusion criteria (1), as well as thyroid ultrasound characteristics and clinical conditions. All FNAB patients signed informed consent forms. This study was conducted in accordance with the Declaration of Helsinki and approved by the Ethics Committee of Aviation General Hospital.

### Inclusion criteria for FNAB

Before FNAB, all patients were graded for thyroid nodules by the attending physician or a higher-level physician in the ultrasound department. One or more of the following malignant signs in the thyroid ultrasound examination indicated an increased risk of malignancy: solid, low echo or very low echo, microcalcification, blurred boundary/differential lobe, aspect ratio >1^A^. The decision of whether to perform FNAB was made based on the features of thyroid nodule ultrasound, medical history, and family history.

### FNAB procedure

Place the patient in the supine position and use a neck pillow to fully expose the patient's neck. To ensure strict aseptic operation for FNAB, disinfect the skin of the neck with iodophor and spread a sterile towel. Insert the needle from the side of the ultrasonic probe under the guidance of ultrasound and direct the whole needle tip to reach the thyroid nodule during the operation, ensuring that the capillary sampling site is at the target nodule. Use a 22G injection needle, a 22G biopsy needle, or a 25G biopsy needle as the capillary sampling needle. Use the vacuum aspiration method when aspirating cells with a 22G injection needle, and use the capillary siphoning method when aspirating cells with a 22G biopsy needle or a 25G biopsy needle. Prepare 1–2 smears using the cells obtained from each biopsy, perform routine biopsy three times at each nodule, and obtain 3–6 samples from each nodule. The samples were considered unsatisfactory if all samples from the same nodule did not meet the diagnostic conditions [7]. All FNAB procedures were performed by a pathologist with more than 10 years of experience under ultrasound guidance.

### Specimen determination

All smear samples were completed by two pathologists with titles of attending physician or higher, and the pathological diagnosis was made based on the “Bethesda system for reporting thyroid cytopathology” (TBSRTC), 1st edition [8]. See the six diagnostic classifications of TBSRTC in Table 1. TBSRTC Class I was the diagnostic unsatisfactory group, while the other five were the satisfactory groups.

**Table 1:** The Bethesda system for reporting thyroid cytopathology (TBSRTC).

Category	Clinical significance	Risk of malignancy (%)
Category I, TBSRTC	Non-diagnosable or unsatisfactory specimens	1–4
Category II, TBSRTC	Benign follicular nodules	0–3
Category III, TBSRTC	Atypical cells of uncertain significance	5–15
Category IV, TBSRTC	Follicular tumor or suspected follicular tumor	15–30
Category V, TBSRTC	Suspected malignant tumor	60–75
Category VI, TBSRTC	Malignant tumor	97–99

### Statistical method

SPSS 20.0 software was used for statistical processing, and the measurement data were expressed by mean ± standard deviation (*x* ± *s*). After a normality test and a test for determining the homogeneity of variances, an independent sample analysis of variance was made to compare the measurement data of normal distribution and homogeneity of variance between the three groups. The enumeration data were expressed by the number of cases and percentage, and the *χ*^2^ test was used to compare the enumeration data among the three groups. The difference was considered statistically significant if *P *< 0.05. Logistic bivariate regression analysis was used to analyze the risk factors, with *α* = 0.05.

## Results

### Demographics and baseline data

A comparison of baseline clinical data among the three groups indicated that there were no statistically significant differences among the three groups in terms of gender ratio, age, and thyroid function (*P *> 0.05), as shown in Table 2. According to the ultrasonic examination features, there were no significant differences among the three groups in terms of nodule blood flow, calcification, and solid-cystic type. The diameter of the thyroid nodules in Group 1 was slightly larger than those in Groups 2 and 3. There were more nodules with an aspect ratio greater than 1 in Group 2.

**Table 2:** Comparison between the two groups in terms of general clinical conditions and thyroid ultrasound features (*x *± *s*).

Group	Group 1	Group 2	Group 3	*P* value
*n* (M/F)	21/64	10/40	48/156	0.817
Age (Years)	48.35 ± 13.48	50.80 ± 13.97	46.15 ± 13.27	0.067
TSH (uiu/ml)	1.94 ± 1.22	1.93 ± 1.39	2.08 ± 1.18	0.628
Nodule size (cm)	1.32 ± 0.87	1.01 ± 0.96	1.07 ± 0.79	0.049
Nodule blood flow (no/little/abundant)	18/43/24	18/23/9	63/106/35	0.139
Nodule type (solid/solid-cystic)	72/13	44/6	188/16	0.15
Aspect ratio greater than 1 (case)	18/85	22/50	73/204	0.012
Calcification (case) (no/tiny/thick)	42/26/17	19/22/9	94/84/26	0.253

Group 1: Vacuum aspiration with 22G injection needle; Group 2: Capillary siphoning with 22G biopsy needle; Group 3: Capillary siphoning with 25G biopsy needle.

### Analysis of the capillary sampling satisfaction rate of the three groups

The dissatisfaction rates of capillary sampling results in the three groups were 22/85 (25.88%), 15/50 (30%), and 18/204 (8.82%), respectively, indicating significant differences among the three groups (*P *< 0.001). The capillary sampling dissatisfaction rate of patients in Group 3 was significantly lower than that of those in the other two groups (see Table 3). There were no significant differences between Groups 1 and 2.

**Table 3:** Comparison between different capillary sampling needles and aspiration methods in terms of cytopathological satisfaction of thyroid fine-needle aspiration biopsy (FNAB).

Pathological results	Group 1	Group 2	Group 3	Chi-square value	*P* value
Satisfactory puncture results	63	35	186	21.04	<0.001
Unsatisfactory puncture results	22	15	18		

Group 1: Vacuum aspiration with 22G injection needle; Group 2: Capillary sampling with 22G biopsy needle; Group 3: Capillary sampling with 25G biopsy needle.

### Logistic regression analysis of independent risk factors for unsatisfactory capillary sampling results

Risk factor analysis: Logistic regression analysis was conducted for age, gender, nodule size, nodule location, nodule blood flow, nodular aspect ratio, nodule morphology, nodule border, nodule calcification, TSH level, and capillary sampling needles and aspiration methods. The statistical results showed that age and capillary sampling needles and aspiration methods were the independent risk factors for thyroid capillary sampling pathological results, with the odds ratio (OR) values being 0.95 and 2.03, respectively (see Table 4).

**Table 4:** Logistic regression analysis of independent risk factors for unsatisfactory thyroid FNAB pathological results.

Index	*B*	SE(*b*)	Wald–*χ*	*P*	OR	95% CI
Age	−0.55	0.013	16.64	<0.001	0.95	0.92–0.97
Gender	0.431	0.405	1.132	.287	1.54	0.70–3.41
Nodule size	−0.086	0.225	0.144	0.704	0.918	0.590–1.428
Nodule location	0.373	0.350	1.137	0.286	1.452	0.731–2.884
Nodule blood flow	0.080	0.274	0.086	0.769	1.084	0.634–1.853
Nodular aspect ratio	0.086	0.406	0.045	0.833	1.090	0.492–2.415
Nodule morphology	0.667	0.451	2.187	0.139	1.948	0.805–4.713
Nodule border	−0.663	0.401	2.731	0.098	0.515	0.235–1.131
Nodule calcification	−0.245	0.264	0.861	0.354	0.783	0.466–1.314
TSH level	−0.041	0.149	0.077	0.781	0.960	0.717–1.285
Capillary sampling needles and aspiration methods	0.699	0.196	12.74	<0.001	2.03	1.37–2.99

OR, odds ratio.

## Discussion

In the current TBSRTC system (2nd edition), guidelines for the management of patients with thyroid nodules, the introduction of molecular testing as an adjunct to cytopathologic examination, and the reclassification of the non-invasive follicular variant of papillary thyroid carcinoma as non-invasive follicular thyroid neoplasm with papillary-like nuclear features are all revised [9]. According to the current system of TBSRTC, thyroid nodules are classified into six diagnostic categories as follows: non-diagnostic (ND) or unsatisfactory (UNS); benign; atypia of undetermined significance (AUS) or follicular lesion of undetermined significance (FLUS); follicular neoplasm (FN) or suspicious for a follicular neoplasm (SFN); suspicious for malignancy (SFM); and malignant. The risks of malignancy for the six categories are 5% to 10%, 0% to 3%, 6% to 18%, 10% to 40%, 45% to 60%, and 94% to 96%, respectively, which are different from TBSRTC, 1st edition (10).

The detectable rate of thyroid nodules in the population is increasing year by year, with continuous improvement in people's health awareness and ultrasound technology, which is 20%–70% in different populations [11–13]. Malignant tumors account for 5%–15% of thyroid nodules [1, 3]. Thyroid tumors are prone to secondary changes, such as calcification, fibrosis, edema, and hemorrhage [6], which provide a basis for distinguishing benign and malignant nodules. Characterized by simplicity, economy, and non-invasiveness, high-resolution ultrasound technology is the preferred examination method for the clinical screening of both benign and malignant thyroid nodules [1, 6]. However, there are obvious overlap manifestations in the ultrasound characteristics of benign and malignant thyroid tumors, due to multiple pathological types and the heterogeneity of the biological characteristics of thyroid cancer. In addition, ultrasound diagnosis depends on the experience of sonologists, the resolution of ultrasound equipment, and other factors, and the coincidence rate of thyroid ultrasound and pathology is 60%–80% [6]. Therefore, in both domestic and foreign guidelines, FNAB is recommended for nodules with malignant signs in thyroid ultrasound imaging. FNAB can improve the preoperative diagnosis accuracy of thyroid nodules by up to 80%–90% [1, 7] and is recognized as the gold standard for determining benign and malignant thyroid nodules.

Individual differences in thyroid nodules and the complexity of clinical conditions can contribute to the failure to make a diagnosis based on the cytopathological results of thyroid FNAB in certain patients, affecting one’s judgment on the nature of thyroid nodules. Previous studies have shown that the factors affecting the unsatisfactory pathological diagnosis of FNAB may include the nature of nodules (such as cystic nodules), the size of the thyroid capillary sampling needle, and the method of cell aspiration [14–16]. After a series of improvements in thyroid capillary sampling needles and aspiration methods, it was found that fine needles are recommended for thyroid capillary sampling for their safety and favorable effect, and 21–27G capillary sampling needles are preferred over others [17]. However, there is still a controversy surrounding which type of capillary sampling needle has the best effect. This is a retrospective study, and the results of logistic bivariate regression analysis suggest that the diameter of the needle and the aspiration method used for thyroid capillary sampling, as well as the age of the patient, are independent risk factors for unsatisfactory pathological diagnosis of FNAB, which is consistent with those of previous studies [14]. The risk coefficients of the two independent risk factors in this study were 2.01 and 0.95, respectively, suggesting that different needle diameters and aspiration methods had a significant influence on the results. According to the Birmingham Wire Gauge, the larger the size of the capillary sampling needle, the smaller the aperture will be. The outer diameters of the 25G biopsy needles and the 22G needles used in this study were 5 and 7 mm, respectively. Through an analysis and comparison of the influences of different needle diameters and aspiration methods on the pathological results, it was found in this study that a 25G needle without vacuum aspiration could increase the FNAB diagnosis rate, which was consistent with previous research results [18], while a 27G capillary sampling needle had also been shown to be better in some studies [19], indicating that the effect of fine needles is better than that of thicker needles. Accumulated studies of the thyroid fine-needle aspiration technique have reported that the sufficiency for diagnosis could be increased by selecting smaller needles [20, 21]. Very recently, Shumrick et al. [22] carried out a study to compare the diagnostic capability between 25G and 27G needles for ultrasound-guided fine-needle biopsy of thyroid nodules, and they concluded that a 27G needle for FNBs yields better diagnostic information than 25G needles. Sengul et al. [23–27] have recommended that Thy MIFNA with a 27G fine-needle could be applied in the ultrasonography-guided FNA procedure due to low rates of Category I (TBSRTC, 1st and 2nd editions.) and low severity of pain. Clinically, the special aspiration biopsy needles for thyroid biopsy are used based on the capillary siphoning principle rather than vacuum aspiration, and capillary siphoning can successfully extract cells, which can reduce bleeding while providing convenience. Gill et al. [14] showed that the non-vacuum aspiration method could significantly reduce the dissatisfaction rate of FNAB pathological diagnosis. By comparing the influence of a 22G needle with different aspiration methods on pathological diagnosis, this study showed that there was no significant difference in the dissatisfaction rate of extracted cells with different aspiration methods, which was inconsistent with the research results of Gill et al. [28] but consistent with the results of previous meta-analysis. The study revealed that compared with a 22G needle, a 25G needle could significantly reduce medical expenses and save medical costs from the perspective of economic benefit and cost ratio, and the effect was similar to that of the use of a 22G biopsy needle. However, there were few cases in the 22G capillary sampling needle group in this study, and there was a lack of comparison with vacuum aspiration because it was not used in the 25G capillary sampling biopsy needle group. Therefore, in order to further prove the causal relationship between the vacuum aspiration and the satisfaction rate of the pathological results of thyroid capillary sampling biopsy, a randomized controlled clinical research study with a large sample size is required for verification.

In conclusion, despite the limited sample size and possible deviations in the sample selection and statistical process, this study suggests that a 25G aspiration biopsy needle without vacuum aspiration could significantly improve the pathological diagnosis rate of FNAB, and that the use or non-use of vacuum aspiration in the 22G capillary sampling needle group had little influence on cytodiagnosis.

## Data Availability

The original contributions presented in the study are included in the article/Supplementary Material, Further inquiries can be directed to the corresponding author.

## Footnotes

^A^
The aspect ratio of thyroid B-mode ultrasound is greater than 1. The concept of aspect ratio refers to the ratio of the anterior to posterior diameter of a lesion or nodule to the left and right diameter on the transverse section of the thyroid gland. The longitudinal refers to the anterior to posterior diameter, and the transverse refers to the left and right diameter. If the anterior and posterior diameter of the nodule is greater than the left and right diameter, and the longitudinal ratio is greater than 1, the nodule tends to be upright on ultrasound images, and such nodule is more likely to be malignant.
